# Attraction to Motion

**DOI:** 10.1371/journal.pbio.0030251

**Published:** 2005-06-07

Anyone with a passing familiarity with animal behavior knows the classic photo of Konrad Lorenz trailed by a gaggle of goslings. Lorenz showed that geese hatched in an incubator identified with the first moving stimulus they saw within 36 hours after birth. When the first thing was Lorenz—or more accurately, his boots—the baby geese imprinted on him.

But what can a newborn recognize? What elements in the visual world are their brains preprogrammed to process? In a new study, Giorgio Vallortigara, Lucia Regolin, and Fabio Marconato take advantage of the natural imprinting behavior of newly hatched chickens to study motion perception. It's clear that animals are more likely to respond to something that moves than to something that doesn't and that the motion of animate objects (biological motion) has uniquely identifiable characteristics. Is this identification innate or is it something an animal learns through experience? [Fig pbio-0030251-g001]

**Figure pbio-0030251-g001:**
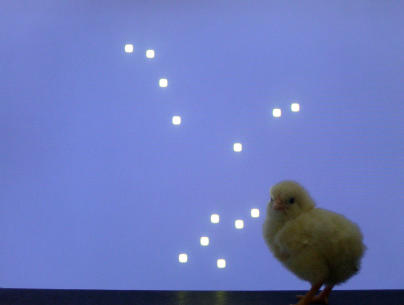
Are you my mom? “Point light animations” test chicks' preference for biological motion

To tackle these questions, the researchers presented chicks with artificially induced motion patterns. The chicks were immediately drawn to and stayed close to iconic patterns of animal motion, also known as biological motion. Interestingly, the chicks didn't distinguish between friend and foe: they were just as likely to approach a cat as a hen.

To test the chicks' preference for biological motion, the researchers took advantage of the fact that many vertebrates—whether they be geese, chickens, or humans—move in a distinctive, coordinated manner. By strategically positioning tiny lights at key points along an animal's torso and limbs, it's possible to strip a moving object of all extraneous traits like shape, texture, and color. People watching such “point light display” animations can easily recognize a person walking, discern the person's gender, and even identify a friend.

Animals are similarly endowed with the ability to extract information from point light displays. To test the chicks' preference for different types of motion, Vallortigara et al. created four animations from 13 points of light. One represented a walking hen, a second created the impression of a “rotating rigid hen-like object” (true animal movement is fluid, though constrained by the skeleton), a third moved in arbitrary directions, and a fourth, the “scrambled hen,” conveyed biological motion, but of an unknown creature (as perceived by human observers). Chicks were hatched in darkness to make sure that the first thing they saw was one of the animations.

The chicks consistently approached the walking and scrambled hen, showing far less affinity for the rigid and random motion, suggesting a predisposition toward the movement typical of vertebrates. As a control, the authors generated an animation of a walking cat. Sure enough, chicks approached the walking cat as often as they approached the walking hen. Luckily for chicks, encounters with cats are not normally likely to precede encounters with a mother hen.

These results suggest that chicks have evolved a predisposition to notice objects that move like vertebrates, which may maximize the probability of imprinting on the object most likely to provide food and protection after birth. This predisposition likely guides the learning that occurs during the imprinting process, when the chick learns how to distinguish mom from other hens, and hens from cats. Since both birds and mammals (tested in four-month-old human infants) show a preference for biological motion, the authors conclude, these results suggest that this preference is hard-wired into the vertebrate brain. With this new model, researchers can investigate the motion-specific features that guide the chicks' behavior and how the brain processes biological motion.

